# Clinical Utility of Pan-Immune Inflammation Value (PIV) in Predicting Prognosis of Endometrial Cancer

**DOI:** 10.3390/jcm14217885

**Published:** 2025-11-06

**Authors:** Nurhan Onal Kalkan, Zuhat Urakcı, Berrak Mermit Erçek, Erkan Bilen, Hayati Arvas, Mehmet Hadi Akkuş

**Affiliations:** 1 Department of Medical Oncology, Batman Training and Research Hospital, 72070 Batman, Turkey; erkanbilen@yahoo.com (E.B.); drmhakkus@gmail.com (M.H.A.); 2 Department of Medical Oncology, Faculty of Medicine, Dicle University, 21280 Diyarbakır, Turkey; dr.zurak@hotmail.com (Z.U.); hayatiarvas65@gmail.com (H.A.); 3 Department of Medical Oncology, Faculty of Medicine, Yuzuncu Yil University, 65090 Van, Turkey; drmermitberrak@gmail.com

**Keywords:** endometrial cancer, PIV, SIRI, SII, systemic inflammation, prognosis

## Abstract

**Background**: Endometrial cancer (EC) is the most common gynecological malignancy in developed countries. While early-stage disease has favorable outcomes, advanced or recurrent EC remains associated with poor prognosis. Novel prognostic markers are needed to refine risk stratification. Systemic inflammation-based indices such as Pan-Immune Inflammation Value (PIV), Systemic Inflammation Response Index (SIRI), and Systemic Immune Inflammation Index (SII) have shown prognostic potential in solid tumors. **Methods**: We retrospectively evaluated 78 patients with endometrioid EC who had undergone hysterectomy with adnexectomy and lymphadenectomy. Demographic, clinicopathological, and laboratory data were extracted from electronic medical records. PIV, SII, and SIRI were calculated from the preoperative complete blood counts. Survival was assessed using Kaplan–Meier analysis, while prognostic factors were determined using univariate and multivariate Cox regression analyses. **Results**: The median age was 59 years, and 64.1% of the patients presented with early-stage disease. A high PIV (≥802) was significantly associated with a shorter overall survival (64 vs. 111 months, *p* < 0.001). PIV demonstrated the highest discriminatory accuracy (AUC = 0.776), followed by the SII (0.747) and SIRI (0.718). Univariate analysis identified that age, grade, LVSI, PNI, stage, distant metastasis, and high PIV, SII, SIRI, and NLR were predictors of poor survival. Multivariate analysis confirmed grade, distant metastasis and SIRI ≥ 1.5 as independent prognostic factors. **Conclusions**: Inflammation-based indices, particularly PIV and SIRI, correlated with survival outcomes in patients with EC. The SIRI retained an independent prognostic value, whereas PIV showed a strong discriminatory capacity. Incorporating these indices into established risk models may improve prognostic precision and support individualized management.

## 1. Introduction

Endometrial cancer (EC) is the most prevalent gynecological malignancy and its incidence is increasing in developed countries [1]. In 2020, 417,336 women worldwide were diagnosed with EC, making it the sixth most common cancer in women [2]. Although early diagnosis is associated with favorable outcomes, the prognosis remains poor for women diagnosed at later stages or with recurrent or metastatic disease [3]. Endometrial carcinoma accounts for approximately 90% of all uterine cancers, and surgical intervention with or without adjuvant radiotherapy or chemotherapy remains the primary therapeutic approach [4].

Histologically, EC is classified into two main forms: endometrioid (type I), which represents approximately 80% of EC cases, and non-endometrioid (type II), which accounts for the remaining 20% [5]. More recently, molecular classification has further subdivided EC into four groups: EC with polymerase epsilon mutations (POLEmut), EC with mismatch repair deficiency (dMMR), high microsatellite instability (MSI-high), EC with TP53 mutations and abnormal p53 protein expression (p53abn), and EC without a specific molecular profile (NSMP) [6]. Prognosis and therapeutic strategies vary by molecular subtype, with POLEmut tumors generally associated with excellent outcomes and p53abn tumors associated with the poorest prognosis. Accordingly, subtype-specific adjuvant treatments, including targeted therapy and immunotherapy, are increasingly being recommended [7,8]

Although the majority of women with EC achieve favorable outcomes, a notable proportion present with adverse clinicopathological features, indicating poor prognosis [9]. Numerous studies have sought to identify prognostic factors for EC, as the early detection of recurrence with reliable markers may improve treatment outcomes and survival. Established prognostic variables include histologic subtype, tumor grade, lymphovascular space invasion (LVSI), and International Federation of Gynecology and Obstetrics (FIGO) staging [10,11]. However, survival outcomes may vary considerably even among patients with similar established prognostic profiles. This highlights the need to identify novel and more specific biomarkers to improve risk stratification and guide personalized treatment.

Blood-based biomarkers, particularly inflammatory markers, have shown promise for predicting the prognosis of EC [12]. Peripheral blood testing is a convenient and cost-effective method for assessing high-risk patients. Determining the prognostic status at diagnosis may allow for more tailored treatment strategies and potentially improve survival outcomes [13]. Inflammation and immune response play pivotal roles in carcinogenesis, including initiation, invasion, promotion, and metastasis [14]. The interplay between tumor cells and components of the tumor microenvironment, such as neutrophils, lymphocytes, platelets, and monocytes, is thought to drive tumor proliferation and metastasis [15]

Several inflammatory indices derived from routine blood counts, including the Pan-Immune Inflammation Value (PIV), Systemic Inflammatory Response Index (SIRI), and Systemic Immune Inflammation Index (SII), have been associated with poor prognosis in various solid tumors [16,17,18,19]. This correlation is attributed, in part, to the neutrophil-mediated secretion of growth factors such as Vascular Endothelial Growth Factor (VEGF) and proteases that promote metastasis [20]. Conversely, reduced lymphocyte counts may compromise antitumor immunity by impairing cytotoxic cell function and diminishing tumor cell clearance [21].

In this context, our retrospective analysis aimed to evaluate the relationship between peripheral immune cell count and distant metastases in patients with EC. We propose that inflammatory nomograms based on such indices may represent promising novel prognostic markers that complement and potentially surpass traditional staging systems in predicting outcomes and guiding personalized management strategies for advanced gynecological malignancies.

## 2. Materials and Methods

Study Population: Between 1 January 2015 and 1 January 2023, the clinicopathological data of 150 patients diagnosed with endometrial carcinoma (EC) at our institution were retrospectively collected. Eligible patients underwent hysterectomy with adnexectomy and lymphadenectomy. Preoperative evaluation included gynecological examination, imaging studies, and histopathological assessment.

**Figure 1:**
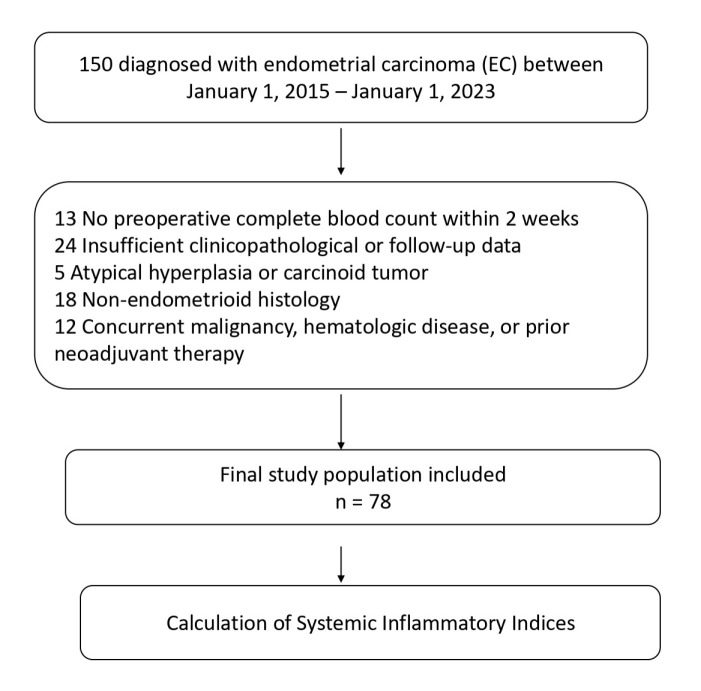
Flowchart of the study according to CONSORT diagram.

Exclusion criteria were as follows: 13 patients without a complete blood count within two weeks prior to surgery, 24 patients with insufficient clinicopathological or follow-up data, 5 patients with atypical hyperplasia or carcinoid tumors, 18 patients with non-endometrioid histology, and 12 patients with concurrent malignancies, hematologic disorders, or a history of neoadjuvant therapy. After applying these criteria, 78 patients were included in the final analysis. Comprehensive biochemical evaluations, including complete blood count, were performed within 15 days prior to surgical intervention. Patients with chronic immune or inflammatory disorders, active acute infections, a recent history (within the past month) of medications affecting immune or inflammatory responses (Figure 1).

### 2.1. Data Collection

Clinical data were obtained from the hospital’s archived electronic medical records. Demographic variables included age, menopausal status, comorbidities, smoking status, and obesity (BMI ≥ 30 kg/m^2^). Pathological parameters included tumor grade, perineural invasion (PNI), lymphovascular space invasion (LVSI), pathological T stage (pT), presence of distant metastasis, and overall stage. Treatment-related variables included administration of adjuvant chemotherapy and radiotherapy. Progression-free survival (PFS) was defined as the interval between the date of pathological diagnosis and the occurrence of disease progression, death, or the last follow-up visit. Overall survival (OS) was defined as the time from the date of pathological diagnosis to death from any cause or the last documented follow-up.

#### Calculation of Systemic Inflammatory Indices

Peripheral blood samples obtained at baseline were analyzed to determine neutrophil, platelet, monocyte, and lymphocyte counts (expressed as ×10^3^/mm^3^). Based on these parameters, systemic inflammatory indices were calculated according to previously validated formulas. The systemic immune inflammation index (SII) was defined as neutrophil count × platelet count/lymphocyte count. The systemic inflammation response index (SIRI) was calculated as neutrophil count × monocyte count/lymphocyte count. The pan-immune inflammation value (PIV) was determined as neutrophil count × platelet count × monocyte count/lymphocyte count.

This study was conducted in accordance with the Declaration of Helsinki (revised in 2024). Ethical approval was obtained from the Ethics Committee of Batman Training and Research Hospital, Non-Interventional Clinical Studies, dated 23 January 2025, decision no: 406.

### 2.2. Statistical Analysis

Statistical analyses were performed using the Statistical Package for the Social Sciences (SPSS) for Windows version 27 (IBM SPSS Inc., Chicago, IL, USA). The normality of the data distribution was assessed using the Kolmogorov–Smirnov test. Continuous variables with normal distribution are expressed as mean ± standard deviation, whereas non-normally distributed variables are presented as medians (min–max).

The diagnostic performance of PIV, SII, SIRI, NLR, and PLR in predicting mortality was evaluated using receiver operating characteristic (ROC) curve analysis. The DeLong method was used to calculate the confidence intervals in the AUC table. The optimal cut-off values for these indices were determined using the Youden index method. Associations between PIV and clinicopathological and laboratory parameters were assessed using the chi-square, Mann–Whitney U, as appropriate.

Overall survival (OS) and progression-free survival (PFS) were estimated using the Kaplan–Meier method and compared using the log-rank test. The potential for multicollinearity was assessed by calculating the Variance Inflation Factor (VIF) values for the inflammatory indices included in the regression model. The VIF values ranged from 0.6 to 3.8, no significant multicollinearity was detected. Therefore, all variables were retained in the multivariate regression analysis. The impact of the variables on survival outcomes was further analyzed using univariate and multivariate Cox proportional hazard regression models. Statistical significance was set at a two-tailed *p*-value of <0.05.

## 3. Results

A total of 78 patients with endometrioid carcinoma were included in this study. The median age of the patients was 59 years (range, 24–91 years), and 30 patients (38.5%) were aged ≥65 years. Twenty-eight patients (35.9%) were premenopausal, and 50 (64.1%) were postmenopausal. The ECOG performance status was 0–1 in 65 patients (83.3%) and ≥2 in 13 patients (16.7%). Nineteen patients (24.4%) were smokers, 32 (41.0%) were obese, and 49 (62.8%) had comorbidities. The histological grade was 1 in 32 patients (41.0%), 2 in 17 patients (21.8%), and 3 in 29 patients (37.2%). LVSI was present in 21 patients (26.9%), and PNI in 10 patients (12.8%). Adjuvant radiotherapy and chemotherapy were administered to 36 (46.2%) and 27 (34.6%) patients, respectively. Peritoneal involvement was detected in 13 patients (16.7%), and distant metastases in 15 patients (19.2%). According to risk stratification, 47 (60.3%) were low-risk and 31 (39.7%) were high-risk. Nodal involvement was observed in 19 (24.4%) patients. pT1–2 was present in 50 (64.1%) and pT3–4 in 28 (35.9%). Early-stage disease (I–II) was identified in 50 patients (64.1%) and advanced stage disease (III–IV) in 28 patients (35.9%).

**Figure 2:**
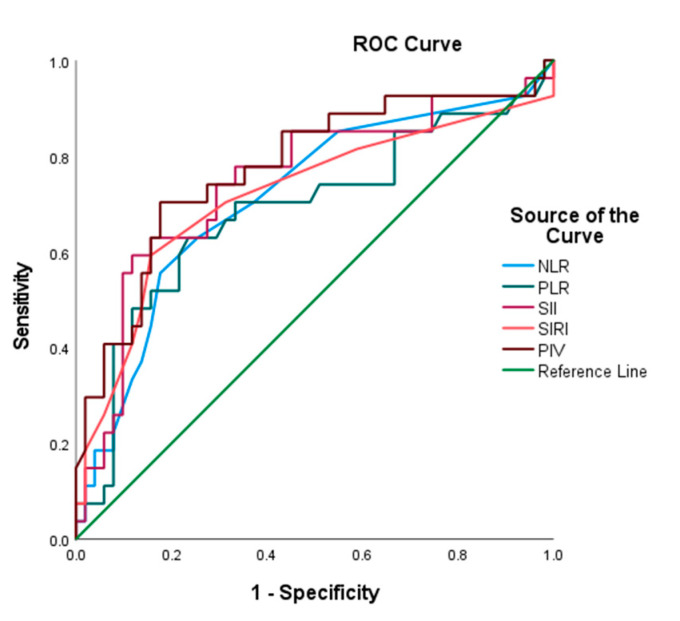
ROC curves for systemic inflammation indices in predicting mortality of endometrial cancer patients.

ROC analysis demonstrated that PIV had the highest prognostic accuracy (AUC = 0.776, sensitivity = 70.4%; specificity, 82.4%; cut-off, 802). SII (AUC = 0.747), SIRI (AUC = 0.718), and NLR (AUC = 0.713) also showed significant discriminatory values, whereas PLR had a lower predictive ability (AUC = 0.686) (Figure 2, Table 1).

Fifty patients (64.1%) had low PIV (<802) and 28 (35.9%) had high PIV (≥802). SII was <1545 in 53 (67.9%) and ≥1545 in 25 (32.1%). SIRI was <1.5 in 43 (55.1%) and ≥1.5 in 35 (44.9%). NLR was <2.5 in 38 (48.7%) and ≥2.5 in 40 (51.3%). PLR was <120.5 in 22 (28.2%) and ≥120.5 in 56 (71.8%). High PIV was significantly associated with obesity (*p* = 0.029), PNI (*p* = 0.022), and adjuvant radiotherapy (*p* = 0.015) but not with age, menopausal status, ECOG status, smoking, comorbidity, tumor grade, LVSI, chemotherapy, peritoneal involvement, metastasis, risk group, nodal involvement, pT, or FIGO stage. Elevated PIV was strongly correlated with other inflammation-based indices, including SII, SIRI, NLR, and PLR (all *p* < 0.001) (Table 2).

**Figure 3:**
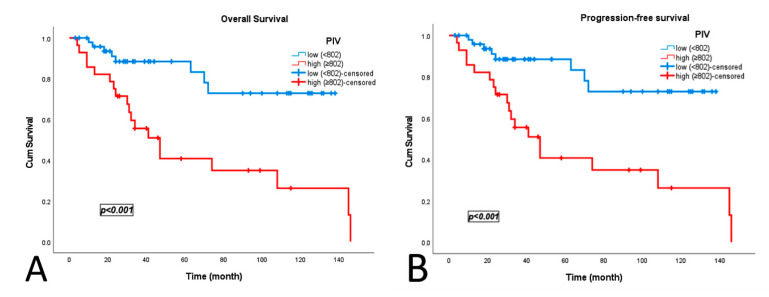
Kaplan–Meier survival curves according to Pan-Immune Inflammation Value (PIV): (**A**) overall survival; (**B**) progression-free survival.

During a median follow-up period of 87 months, 27 patients (34.6%) died. The median overall survival (OS) was 95.5 months (range, 34.2–146), with significantly longer survival in the low PIV group (111 months [96.0–127.5]) than in the high PIV group (64 months [43.4–85.2], *p* < 0.001). Disease progression occurred in 24 patients (30.8%), with a median progression-free survival (PFS) of 80 months (range, 43.2–106.6). Median PFS was longer in the low PIV group (105 months [88.5–121.0]) than in the high PIV group (90 months [64.6–116.0]), though the difference was not statistically significant (*p* = 0.446; Figure 3).

In the univariate Cox regression analysis, advanced age (≥65 years), higher grade, LVSI, PNI, adjuvant chemotherapy, distant metastasis, high-risk classification, advanced pT and stage, and elevated inflammatory indices (PIV ≥ 802, SII ≥ 1545, SIRI ≥ 1.5, NLR ≥ 2.5) were significantly associated with poorer overall survival (OS), while obesity emerged as a protective factor. PLR ≥ 120.5 demonstrated borderline significance. For progression-free survival (PFS), obesity remained protective, whereas grade, LVSI, PNI, chemotherapy, metastasis, high-risk status, advanced pT, stage, and the same inflammatory indices were significant adverse prognostic indicators, with PLR not reaching significance (Table 3).

In the multivariate Cox regression analysis, distant metastasis, higher tumor grade, and elevated SIRI remained independent poor prognostic factors for overall survival, while PIV, SII, and NLR were not significant. For progression-free survival, grade, dis-tant metastasis, and SIRI were also confirmed as independent predictors. PIV and NLR showed a trend toward increased risk but did not reach statistical significance. No significant associations were observed for age, obesity, LVSI, PNI, adjuvant chemotherapy, risk group, pT, or FIGO stage (Table 4).

## 4. Discussion

Endometrial carcinoma is the most common gynecological malignancy in developed countries. Because of its heterogeneous biological behavior, accurate identification of prognostic factors is of critical importance for clinical management. Traditional prognostic parameters include tumor stage, histological grade, lymphovascular space invasion (LVSI), and perineural invasion (PNI). However, in recent years, there has been growing evidence regarding the prognostic value of biomarkers that reflect systemic inflammatory responses [22]. In this context, our study comprehensively evaluated the prognostic relevance of the Pan-Immune Inflammation Value (PIV), along with SII, SIRI, NLR, and PLR, in a cohort of patients with endometrioid-type endometrial carcinoma.

Our findings revealed that PIV is the strongest predictor of patient survival. In the ROC analysis, PIV achieved the highest AUC (0.776), and patients with values above the cutoff (802) exhibited significantly shorter overall survival. The median OS was 111 and 64 months in the low-and high-PIV groups, respectively (*p* < 0.001). This suggests that PIV, by reflecting the magnitude of the systemic inflammatory response and immune cell interactions within the tumor microenvironment, may serve as a powerful prognostic indicator. Previous studies have also shown that elevated PIV predicts poor outcomes in several solid tumors, particularly ovarian and cervical cancers [22,23,24]. Recently published data similarly reported that high PIV levels were significantly associated with reduced survival, supporting the validity of our findings in endometrial carcinoma [25].

In our cohort, PIV showed no significant association with demographic or clinicopathologic variables, including age, menopausal status, ECOG score, comorbidities, tumor grade, LVSI, chemotherapy, peritoneal or nodal involvement, metastasis, or FIGO stage. This pattern suggests that PIV may primarily reflect systemic inflammatory status rather than intrinsic tumor characteristics. While this supports its potential as an independent prognostic biomarker, it also underscores the importance of integrating inflammatory indices with pathological parameters to achieve a more comprehensive assessment of tumor aggressiveness.

SII, SIRI, and NLR were also identified as significant predictors of OS and PFS in the univariate analyses. These results are consistent with those of previous reports, where high SII values correlated with advanced stage and poor prognosis in endometrial cancer [10,26]. Likewise, the prognostic value of NLR has been documented across various gynecological malignancies, with elevated NLR reflecting immune dysfunction and tumor progression [27]. SIRI has been less extensively studied. Tu et al. reported that SIRI values above 1.29 were significantly associated with an increased risk of recurrence and death in patients with endometrial cancer, whereas those in the low-SIRI group demonstrated longer recurrence-free and overall survival, even in the absence of adjuvant therapy. Similarly, in our cohort, a high SIRI was associated with poorer survival and shorter recurrence intervals, and it retained independent prognostic significance for both overall survival (OS) and progression-free survival (PFS) in multivariate analysis [28]. These findings support the potential of SIRI as a strong and reliable prognostic biomarker for endometrial carcinoma.

In contrast, PLR demonstrated only borderline statistical significance in the present study. While this may suggest a potential role of platelets in tumor progression, PLR appears to be a weaker marker than the other indices. Meta-analyses have similarly highlighted the heterogeneity in the prognostic significance of PLR across tumor types [29].

Interestingly, obesity was more common in the low-PIV group. While obesity is a well-established risk factor for endometrial carcinoma, its inverse association with inflammatory indices may reflect the complex interplay between obesity-driven chronic inflammation and systemic immune response [30].

In multivariate Cox regression analysis, only histological grade, distant metastasis, and SIRI ≥ 1.5 remained as independent prognostic factors. This finding reinforces the importance of established histopathological parameters, suggesting that inflammatory indices may serve as complementary, rather than standalone, prognostic tools. Similarly to our results, Huang et al. reported that the prognostic value of inflammatory indices was particularly evident in advanced-stage and high-risk patients [31]. Therefore, PIV and related indices may contribute to risk stratification models, aiding individualized patient management.

This study has certain limitations that should be acknowledged. The retrospective design, relatively small sample size, and single-center nature may limit the generalizability of the results. In addition, blood-derived inflammatory indices can be influenced by comorbidities, obesity, and concurrent infections, which may confound their prognostic interpretation.

Another important limitation is the absence of molecular classification data (e.g., POLEmut, dMMR, p53abn, NSMP) within the study cohort. Although the introduction provides an overview of the contemporary molecular taxonomy of endometrial carcinoma, these molecular subgroups were not available in our dataset and therefore could not be analyzed. In the current era of molecularly guided risk stratification, the lack of such data limits the ability to assess potential interactions between inflammation-based indices and established molecular profiles.

Therefore, future large-scale, prospective, multicenter studies integrating molecular and systemic inflammation parameters are warranted to validate and extend the prognostic value of these indices in endometrial carcinoma. 

## 5. Conclusions

In conclusion, our study demonstrated that inflammatory indices, particularly PIV, may have prognostic value in endometrial carcinoma. Elevated PIV was associated with shorter survival, whereas SIRI was an independent predictor of both OS and PFS. These findings suggested that inflammatory markers should be considered in conjunction with conventional histopathological factors. The incorporation of such indices into future risk stratification models may support more personalized treatment and follow-up strategies for patients with endometrial carcinoma.

## Figures and Tables

**Table 1:** Predictive performance of systemic inflammatory indices for mortality according to ROC analysis.

	AUC	Std. Error	95% CI	Sensitivity	Specificity	Cut-Off Value	*p*
PIV	0.776	0.06	0.659–0.893	70.4%	82.4%	802	0.001
SII	0.747	0.063	0.623–0.870	63%	74.5%	1545	0.001
SIRI	0.718	0.068	0.585–0.850	81.5%	41.2%	1.20	0.002
NLR	0.713	0.064	0.587–0.839	85.2%	45.1%	2.50	0.002
PLR	0.686	0.069	0.551–0.820	85.2%	32.3%	120.5	0.007

Abbreviations: AUC, area under the curve; PIV, Pan-Immune Inflammation Value; SII, Systemic Immune Inflammation Index; SIRI, Systemic Inflammation Response Index; NLR, Neutrophil-to-Lymphocyte Ratio; PLR, Platelet-to-Lymphocyte Ratio.

**Table 2:** Association between PIV and clinicopathological variables in endometrial carcinoma.

Variable	Category	Total (n, %)	PIV < 802 (n, %)	PIV ≥ 802 (n, %)	*p*
**Age**	<65	48 (61.5)	33 (66.0)	15 (53.6)	0.200
	≥65	30 (38.5)	17 (34.0)	13 (46.4)	
**Menopausal status**	Premenopausal	28 (35.9)	18 (36.0)	10 (35.7)	0.590
	Postmenopausal	50 (64.1)	32 (64.0)	18 (64.3)	
**ECOG PS**	0–1	65 (83.3)	44 (88.0)	21 (75.0)	0.124
	≥2	13 (16.7)	6 (12.0)	7 (25.0)	
**Smoking**	No	59 (75.6)	38 (76.0)	21 (75.0)	0.564
	Yes	19 (24.4)	12 (24.0)	7 (25.0)	
**Obesity**	No	46 (59.0)	25 (50.0)	21 (75.0)	0.029
	Yes	32 (41.0)	25 (50.0)	7 (25.0)	
**Comorbidity**	No	29 (37.2)	18 (36.0)	11 (39.3)	0.480
	Yes	49 (62.8)	32 (64.0)	17 (60.7)	
**Grade**	1	32 (41.0)	22 (44.0)	10 (35.7)	
	2	17 (21.8)	10 (20.0)	7 (25.0)	0.581
	3	29 (37.2)	18 (36.0)	11 (39.3)	
**FIGO stage**	I–II	50 (64.1)	33 (66)	17 (60.7)	0.410
	III–IV	28 (35.9)	17 (34)	11 (39.3)	
**LVSI**	Absent	57 (73.1)	37 (74.0)	20 (71.4)	0.503
	Present	21 (26.9)	13 (26.0)	8 (28.6)	
**PNI**	Absent	68 (87.2)	47 (94.0)	21 (75.0)	0.022
	Present	10 (12.8)	3 (6.0)	7 (25.0)	
**Adjuvant RT**	No	42 (53.8)	32 (64.0)	10 (35.7)	0.015
	Yes	36(46.2)	18 (36.0)	18 (64.3)	
**Adjuvant CT**	No	51 (65.4)	35 (70.0)	16 (57.1)	0.185
	Yes	27 (34.6)	15 (30.0)	12 (42.9)	
**Peritoneal involvement**	Absent	65 (83.3)	42 (84.0)	23 (82.1)	0.534
	Present	13 (16.7)	8 (16.0)	5 (17.9)	
**Distant metastasis**	Absent	63 (80.8)	40 (80.0)	23 (82.1)	0.535
	Present	15 (19.2)	10 (20.0)	5 (17.9)	
**Risk group**	Low	47 (60.3)	32 (64.0)	15 (53.6)	0.253
	High	31 (39.7)	18 (36.0)	13 (46.4)	
**Nodal involvement**	Absent	59 (75.6)	40 (80.0)	19 (67.9)	0.177
	Present	19 (24.4)	10 (20.0)	9 (32.1)	
**pT stage**	1–2	50 (64.1)	33 (66.0)	17 (60.7)	0.786
	3–4	28 (35.9)	17 (34.0)	11 (39.3)	
**Stage**	I–II	50 (64.1)	33 (66.0)	17 (60.7)	0.410
	III–IV	28 (35.9)	17 (34.0)	11 (39.3)	
**SII**	<1545	53(67.9)	50 (100.0)	3 (10.7)	<0.001
	≥1545	25 (22.1)	0 (0.0)	25 (89.3)	
**SIRI**	<1.5	43 (55.1)	42 (84.0)	1 (3.6)	<0.001
	≥1.5	35 (44.9)	8 (16.0)	27 (96.4)	
**NLR**	<2.5	38 (48.7)	37 (74.0)	1 (3.6)	<0.001
	≥2.5	40 (51.3)	13 (26.0)	27 (96.4)	
**PLR**	<120.5	22 (28.2)	21 (42.0)	1 (3.6)	<0.001
	≥120.5	56 (71.8)	29 (58.0)	27 (96.4)	

Abbreviations: ECOG PS, Eastern Cooperative Oncology Group Performance Status; LVSI, lymphovascular space invasion; PNI, perineural invasion; RT, radiotherapy; CT, chemotherapy; pT, pathological T stage; SII, Systemic Immune Inflammation Index; SIRI, Systemic Inflammation Response Index; NLR, Neutrophil-to-Lymphocyte Ratio; PLR, Platelet-to-Lymphocyte Ratio; PIV, Pan-Immune Inflammation Value.

**Table 3:** Univariate Cox regression analysis of prognostic factors for overall survival and progression-free survival.

	Overall Survival			Progression-Free Survival		
	HR	95% CI	*p*	HR	95% CI	*p*
Age (≥65 vs. <65)	2.26	1.04–4.93	0.040	1.98	0.89–4.38	0.090
Menopausal status	0.72	0.33–1.57	0.409	0.66	0.30–1.47	0.313
ECOG (≥2 vs. 0–1)	0.90	0.34–2.39	0.825	0.73	0.25–2.17	0.578
Smoking (Yes vs. No)	0.83	0.31–2.19	0.703	0.87	0.33–2.33	0.787
Obesity (Yes vs. No)	0.35	0.14–0.86	0.023	0.38	0.16–0.95	0.038
Comorbidity (Yes vs. No)	1.35	0.61–2.99	0.457	1.21	0.54–2.72	0.634
Tumor grade (2–3 vs. 1)	2.07	1.28–3.33	0.003	2.07	1.29–3.34	0.003
LVSI (Present vs. Absent)	3.12	1.38–7.03	0.006	2.85	1.26–6.47	0.012
PNI (Present vs. Absent)	3.89	1.53–9.94	0.004	3.27	1.25–8.59	0.016
Adjuvant RT (Yes vs. No)	1.56	0.72–3.41	0.262	1.50	0.69–3.29	0.301
Adjuvant CT (Yes vs. No)	4.04	1.82–8.99	0.001	3.58	1.62–7.93	0.002
Peritoneal involvement	2.28	0.91–5.75	0.080	2.39	0.95–6.06	0.065
Distant metastasis	2.55	1.06–6.16	0.037	2.48	1.02–6.01	0.044
Risk group (High vs. Low)	4.83	2.14–10.86	<0.001	4.35	1.93–9.79	<0.001
Nodal involvement	2.18	0.93–5.08	0.072	1.89	0.81–4.44	0.144
Pathological T stage (pT3–4 vs. pT1–2)	2.41	1.23–4.71	0.010	2.26	1.16–4.44	0.017
FIGO stage (III–IV vs. I–II)	3.08	1.41–6.71	0.005	2.84	1.30–6.21	0.009
PIV ≥ 802	4.54	1.96–10.47	<0.001	4.02	1.73–9.32	0.001
SII ≥ 1545	3.65	1.65–8.05	0.001	3.28	1.47–7.30	0.004
SIRI ≥ 1.5	3.37	1.46–7.79	0.004	2.94	1.26–6.82	0.012
NLR ≥ 2.5	4.03	1.60–10.10	0.003	3.65	1.45–9.17	0.006
PLR ≥ 120.5	2.43	0.91–6.48	0.077	2.22	0.83–5.96	0.113

Abbreviations: HR, hazard ratio; CI, confidence interval; ECOG, Eastern Cooperative Oncology Group; LVSI, lymphovascular space invasion; PNI, perineural invasion; RT, radiotherapy; CT, chemotherapy; pT, pathological T stage; FIGO, International Federation of Gynecology and Obstetrics; PIV, Pan-Immune Inflammation Value; SII, Systemic Immune Inflammation Index; SIRI, Systemic Inflammation Response Index; NLR, Neutrophil-to-Lymphocyte Ratio; PLR, Platelet-to-Lymphocyte Ratio.

**Table 4:** Multivariate Cox regression analysis of prognostic factors for overall survival and progression-free survival.

	Overall Survival			Progression-Free Survival		
	HR	95% CI	*p*	HR	95% CI	*p*
Age (≥65 vs. <65)	1.97	0.74–5.21	0.172	-	-	-
Obesity (Yes vs. No)	0.82	0.28–2.34	0.704	0.71	0.25–2.06	0.533
Tumor grade (2–3 vs. 1)	1.91	1.01–3.61	0.046	1.91	1.03–3.55	0.040
LVSI (Present vs. Absent)	0.49	0.09–2.57	0.398	0.50	0.10–2.37	0.380
PNI (Present vs. Absent)	0.50	0.10–2.39	0.385	0.62	0.15–2.66	0.524
Adjuvant CT (Yes vs. No)	2.82	0.75–10.58	0.125	2.21	0.62–7.83	0.220
Distant metastasis	5.28	1.47–19.01	0.011	3.40	1.05–11.02	0.042
Risk group (High vs. Low)	1.29	0.15–10.81	0.815	1.73	0.26–11.53	0.574
Pathological T stage (pT3–4 vs. pT1–2)	1.80	0.13–3.26	0.716	0.67	0.12–1.78	0.620
FIGO stage (III–IV vs. I–II)	1.93	0.00–3.50	0.640	1.88	0.01–1.95	0.490
PIV ≥ 802	5.32	0.49–57.10	0.168	4.58	0.46–45.65	0.195
SII ≥ 1545	1.80	0.34–16.08	0.580	1.19	0.32–11.20	0.245
SIRI ≥ 1.5	1.09	0.09–0.97	0.042	1.12	0.08–1.24	0.036
NLR ≥ 2.5	3.81	0.67–21.83	0.133	4.50	0.82–30.36	0.080

Abbreviations: HR, hazard ratio; CI, confidence interval; LVSI, lymphovascular space invasion; PNI, perineural invasion; CT, chemotherapy; pT, pathological T stage; FIGO, International Federation of Gynecology and Obstetrics; PIV, Pan-Immune Inflammation Value; SII, Systemic Immune Inflammation Index; SIRI, Systemic Inflammation Response Index; NLR, Neutrophil-to-Lymphocyte Ratio.

## Data Availability

The datasets used in this study can be made available by the corresponding author upon reasonable request, with permission from the Batman Training and Research Hospital.

## Abbreviations

EC	Endometrial Cancer
PIV	Pan-Immune Inflammation Value
SII	Systemic Immune Inflammation Index
SIRI	Systemic Inflammation Response Index
NLR	Neutrophil-to-Lymphocyte Ratio
PLR	Platelet-to-Lymphocyte Ratio
OS	Overall Survival
PFS	Progression-Free Survival
ROC	Receiver Operating Characteristic
AUC	Area Under the Curve
LVSI	Lymphovascular Space Invasion
PNI	Perineural Invasion
FIGO	International Federation of Gynecology and Obstetrics
ECOG PS	Eastern Cooperative Oncology Group Performance Status
BMI	Body Mass Index
RT	Radiotherapy
CT	Chemotherapy
pT	Pathological T Stage
VEGF	Vascular Endothelial Growth Factor
POLEmut	Polymerase Epsilon Mutated
dMMR	Deficient Mismatch Repair
MSI-high	High Microsatellite Instability
p53abn	Abnormal p53 Expression
NSMP	No Specific Molecular Profile
CI	Confidence Interval
HR	Hazard Ratio
SPSS	Statistical Package for the Social Sciences
